# A Comparison of Radiation and Alkylator-Based Conditioning Therapy Regimens for Allogeneic Stem Cell Transplantation in Acute Myeloid Leukemia: A Clinician’s Perspective

**DOI:** 10.3390/curroncol32070381

**Published:** 2025-07-01

**Authors:** Alejandro Marinos Velarde, Julio Alvarenga Thiebaud, Yazan Madanat, Amir Toor

**Affiliations:** Department of Internal Medicine, University of Texas Southwestern Medical Center, Dallas, TX 75390, USA; julio.alvarengathiebaud@utsouthwestern.edu (J.A.T.); yazan.madanat@utsouthwestern.edu (Y.M.); amir.toor@utsouthwestern.edu (A.T.)

**Keywords:** allogeneic stem cell transplantation, acute myeloid leukemia, conditioning, myeloablative, reduced intensity conditioning

## Abstract

Hematopoietic stem cell transplantation (HSCT) remains the only curative treatment option for most patients with acute myeloid leukemia. Optimizing the conditioning regimen is critical in HSCT planning. Myeloablative conditioning regimens have been shown to reduce relapse but often lead to increased toxicity and non-relapse mortality. In contrast, reduced-intensity conditioning offers a less toxic alternative, though it has been associated with a higher risk of relapse. Emerging conditioning strategies provide a promising balance between reducing toxicity and improving survival outcomes. In this paper, we summarize current available data related to conditioning regimens for patients with AML undergoing HSCT, highlighting novel findings and focusing on emerging modalities.

## Simple Summary

In this article, we discuss different conditioning regimes used for stem cell transplantation in acute myeloid leukemia. Conditioning allows for the reduction of the leukemic burden and prepares the body for the donor’s stem cells. Myeloablative regimens lower relapse chances but come with a higher risk of side effects. Reduced-intensity regimens are less toxic but may result in increased rates of relapse. Identifying conditioning regimens that result in low toxicity but are effective is an area of research interest in the stem cell transplant field. We give an overview of commonly employed conditioning strategies and comment on emerging strategies.

## 1. Introduction

Despite significant advancements in the treatment of acute myeloid leukemia (AML), hematopoietic stem cell transplantation (HSCT) remains the only curative option for patients with primary refractory AML, those with relapsed disease and for patients who are in first complete remission where the disease has high risk cytogenetic and/or molecular features that increase relapse risk [1,2].

Several important factors to consider when planning HSCT include patient age, medical comorbidities, donor selection, graft-versus-host disease (GVHD) prophylaxis, and the conditioning regimen. HSCT conditioning for AML has twofold purposes: reducing the leukemic burden and ablating the recipient’s immune system to allow the engraftment of the donor’s stem cells [3]. Additionally, an ideal conditioning regimen should minimize toxicity while also reducing the risk of relapse. The paradigm has been that reduced intensity conditioning (RIC) reduces non-relapse mortality while resulting in a higher relapse rate compared to myeloablative conditioning (MAC), particularly for patients with evidence of minimal residual disease (MRD) prior to transplant [4,5]. Nevertheless, ongoing research is exploring conditioning regimens that may enhance the balance between toxicity and relapse risk. This review provides an overview of the commonly used conditioning regimens for patients with AML undergoing hematopoietic stem cell transplantation, focusing on emerging regimens with a better risk/benefit profile.

## 2. Conditioning Regimens by Intensity

The Center for International Blood and Marrow Transplant Research (CIBMTR) consensus defines MAC regimens as those that result in profound myelosuppression within 1–3 weeks and would not result in hematologic recovery without stem cell transplantation; those regimens include total body irradiation (TBI) doses ≥ 5 Gy as a single dose or ≥8 Gy in fractionated doses or busulfan > 8 mg/kg PO or IV. Non-myeloablative (NMA) regimens are defined as those that cause limited cytopenias, with marrow recovery possible without stem cell support. Reduced intensity (RIC) includes regimens that cannot be classified as MAC or NMA [6].

The European Group for Blood and Marrow Transplantation (EBMT) initially introduced the concept of RIC at the allogeneic hematopoietic stem cell transplant workshop in Zurich [7]. Most recently, the EBMT developed the Transplant Conditioning Index (TCI) to standardize and better stratify the intensity of a conditioning regimen based on its components [8]. This model was further validated in a cohort of 4060 patients from the EBMT registry, showing a good correlation of the TCI score with non-relapse mortality (NRM) and relapse risk outside other known factors, such as age, performance status, and organ impairment [9]. Interestingly, the group classified as intermediate showed no difference in relapse rates compared to the high TCI group, suggesting that the index allows for the identification of “reduced toxicity regimens”.

### 2.1. Myeloablative Conditioning

MAC regimens involve the administration of alkylating agents with or without radiation. Standard alkylator-based regimens include busulfan plus cyclophosphamide (Bu/Cy) and busulfan plus fludarabine (Bu/Flu). When radiation is employed, it is usually given along with cyclophosphamide (Cy/TBI).

#### 2.1.1. Radiation-Based Myeloablative Conditioning

Radiation-based conditioning regimens utilize high-energy photons to cause irreparable DNA damage in malignant cells. This damage can occur through direct ionization or indirectly through free radical generation, ultimately leading to cell death. Unlike chemotherapy, radiation therapy is not limited by factors, such as blood flow, drug absorption, metabolism, biodistribution, or clearance kinetics. Additionally, radiation therapy induces immune suppression by actively depleting the recipient’s lymphocyte pool while simultaneously damaging malignant cells. Radiation exposure can lead to cell death and stimulate anti-tumor immunity by promoting the expression of neoantigens, which the newly formed immune system may recognize [10]. Furthermore, clones that are resistant to chemotherapy following acute myeloid leukemia (AML) induction may still respond to radiation treatment [11]. The most commonly utilized radiation-based regimen has been cyclophosphamide in combination with total body irradiation (Cy/TBI), which involves administering 120 mg/kg of cyclophosphamide and 12 Gy of TBI [12]. Different TBI doses ranging from 13.5 to 10 Gy have also been used. Agents, such as busulfan, etoposide [13], cytarabine [14], and fludarabine [15], have also been used in combination with TBI. However, there are limited data directly comparing different TBI-based regimens.

A recent network meta-analysis found no significant difference in overall survival among TBI 8 Gy/Flu, Cy/TBI, and Cy with higher TBI doses [16]. Additionally, retrospective data from the CIBMTR (Center for International Blood and Marrow Transplant Research) examined whether higher doses of radiation lead to better outcomes. The study was not limited to AML and also included patients with acute lymphoblastic leukemia (ALL), chronic myelocytic leukemia, and myelodysplastic syndrome (MDS). Around half of the patients were classified as having intermediate or advanced disease status pre-transplant. While the incidence of relapse decreased (36% with standard 12 Gy TBI and 26% with higher dose, 14 Gy TBI, *p* < 0.001), there was no difference in the five-year overall survival rates (42 vs. 45%, *p* = 0.39) [17]. The authors concluded this was likely due to increased NRM (28% vs. 34%, *p* = 0.02).

**Figure 1:**
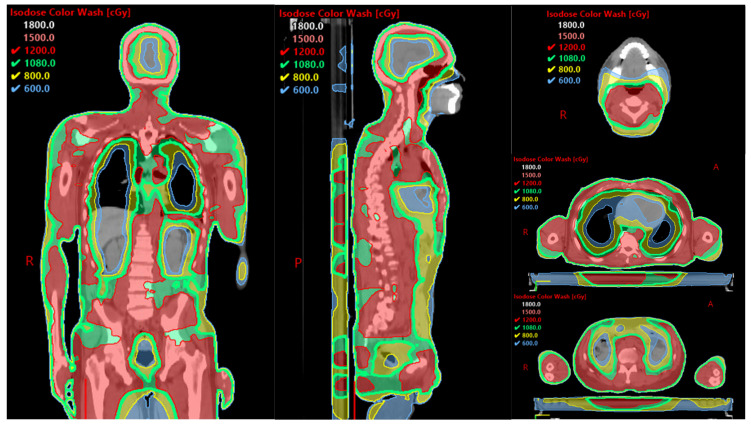
TMLI 15 Gy dose distribution example.

Classical total-body irradiation (TBI) was developed several decades ago and involves the use of opposing whole-body radiation fields. This method delivers a prescribed radiation dose to the entire body but provides minimal protection to normal tissues. As a result, TBI can cause dose differences exceeding 30%, leading to off-target toxicity in non-hematologic tissues due to its broad approach [18]. Furthermore, limiting the radiation dose delivered to the lungs results in lower doses delivered to critical disease sites such as the ribs. Such considerations are of particular importance in obese patients, not only due to the difficulty in adequate planning and issues with treatment delivery, but also the increase in NRM due to the eventual dose being averaged at the midbody plane [19]. In contrast, conditioning regimens utilizing total marrow and lymphoid irradiation (TMLI) employ image-guided, intensity-modulated radiation therapy. TMLI was originally designed for patients with refractory or relapsed diseases who cannot tolerate escalated-dose TBI and experience limited benefits from conventional conditioning. By minimizing radiation exposure to vital organs, TMLI reduces toxicity while allowing myeloablative doses to be delivered to the targeted marrow-bearing regions [20]. Although TMLI may seem more labor-intensive than TBI, the time and resources required for its planning and delivery are comparable. While its application is currently limited to a few centers, TMLI has demonstrated a favorable toxicity profile. This improvement is achieved by identifying at-risk structures, such as the lungs, heart, esophagus/stomach, small and large intestines, kidneys, eyes, lenses, oral cavity, bladder, and parotid glands, aiming to minimize radiation doses delivered to these organs [21]. Consequently, this approach may lead to up to a 60% reduction in radiation dose compared to the standard prescribed dose [22]. (Table 1, Figure 1).

Studies have reported promising results with the use of TMLI. In a phase 1 trial involving the combination of etoposide and cyclophosphamide with dose escalation (12, 13.5, and 15 Gy) of total marrow irradiation (TMI), no dose-limiting toxicities were reported in the highest intensity arm. Conversely, the combination of busulfan plus etoposide and high-dose TMI was not well tolerated; all patients developed grade 3 fatigue and mucositis, but there were no dose-limiting toxicities [13]. The same research group evaluated the tolerability of TMLI at a dose of 20 Gy (twice daily fractions over 5 days) without concurrent alkylating agents in patients 16 to 60 years of age who had AML in either first or second complete remission (CR). A total of 18 patients, 44% of whom had unfavorable risk AML (ELN 2017 or relapsed/refractory), were included in the study. There were no dose-limiting toxicities, the NRM rate was 0% at 2 years, and the overall survival (OS) rate was 86.7%, with a relapse-free survival (RFS) rate of 83.3% [23]. Similar findings have been reported by Zorutti et al. in a series of 10 patients with leukemia (six AML, three acute lymphoblastic leukemia, and one plasma cell leukemia), with no NRM, with a median follow-up of 30 months and a tolerable toxicity profile [24].

#### 2.1.2. Chemotherapy-Based Myeloablative Conditioning

Alkylating agents have the advantage of not being cell-cycle-specific; thus, they are effective in non-proliferating leukemic cells. Several combinations of alkylating agents have been employed for MAC HSCT, with busulfan present in most early regimens. However, busulfan has limited toxicity to mature lymphocytes, which does not allow its use as a single agent, as it does not achieve enough immunosuppression [25]. Santos et al. reported on the use of Busulfan and Cyclophosphamide 200 mg/kg as a radiation-free regimen for HSCT for acute non-lymphocytic leukemia [26]; however, the dose of cyclophosphamide was reduced to 120 mg/kg in a subsequent study [27]. Later, BuFlu4 was described by the MD Anderson Cancer Center HSCT group in 2004 as an alternative to BuCy for myeloablation with a reduced risk for veno-occlusive disease (VOD) [28].

A phase III randomized trial compared Bu/Flu and Bu/Cy in patients with AML, chronic myeloid leukemia, or myelodysplastic syndrome, with fludarabine administered over five days instead of the four days typically used in the more common Bu/Flu4 regimen [16,29]. At the two-year time, the non-relapse mortality (NRM) rates were similar between the two arms, with the Bu/Cy group showing longer overall survival (OS) and relapse-free survival (RFS) rates (67.4% vs. 41.4%, and 74.7% vs. 54.9%, respectively) [30]. In contrast, a meta-analysis of 15 trials involving 1,830 patients found that those receiving Bu/Flu had a lower NRM (RR 0.56) and a lower risk of VOD. There was no significant difference in relapse rates or the 100-day all-cause mortality between Bu/Flu and Bu/Cy [29]. Another, more recent network meta-analysis, which included chemotherapy and total body irradiation (TBI)-based regimens, found no difference in OS between Bu/Flu and Bu/Cy. Nonetheless, it suggested that the Bu3/Flu/Thiotepa regimen (Thiotepa is an alkylating agent with central nervous system penetrance and immunosuppressive activity) may improve OS compared to Bu/Cy (HR 0.70) and that Bu4/Flu has lower NRM compared to Bu/Cy (HR 0.51) [16]. As such, Bu/Flu regimens are commonly thought of as less toxic and as effective as Bu/Cy. It is noteworthy to mention that Bu/Flu is included in the intermediate group by the EMBT-TCI classification (considered a reduced toxicity myeloablative regimens), while Bu/Cy corresponds to the high TCI category [9].

Though IV busulfan has simplified the administration of this drug, it still results in variable exposure when dosed based on weight. Such variability can result in exposure levels that fall outside the intended therapeutic range, which has been linked to an increased rate of relapse, higher NRM, and increased risk of VOD. Furthermore, a randomized controlled trial showed improved OS and PFS when therapeutic drug monitoring (TDM) was used for BuFlu4 in patients with MDS and AML [31]. Briefly, for busulfan TDM, an initial dose is chosen, and then sequential blood draws are obtained to calculate busulfan clearance and, therefore, drug exposure. The appropriate frequency for sampling depends on the frequency of busulfan dosing. Further doses are adjusted based on the calculated busulfan exposure and the total target dose [32]. In 2016, the American Society for Blood and Marrow Transplantation released a Frequently Asked Questions document instead of formal guidelines, noting the variability in the existing literature. Their position is that TDM should be implemented for conditioning regimens where there is a pharmacodynamic correlation between busulfan exposure and clinical outcomes or where data exist showing that busulfan TDM leads to improved outcomes [33].

Older patients or those with comorbidities are not eligible for busulfan MAC regimens due to their increased NRM and commonly receive RIC regimens. Nevertheless, RIC has been associated with a higher risk of relapse. Therefore, there has been interest in developing busulfan MAC protocols with reduced toxicity. Popat et al. have pioneered “fractionated busulfan,” in which the drug is administered in a sequential manner rather than the usual concurrent four days. A phase 2 trial in which adult patients with AML or MDS, which compared two fractionation schedules with either busulfan dosed on days −20, −13, and from days −6 to −3 or a shorter schedule with dosing on days −13 and days −6 to −3, reported grade ≥ 3 toxicities of 73.7% versus 93.2% (*p* = 0.005). The three-year NRM was 7% versus 19%, favoring longer fractionation (*p* = 0.067). There was no difference in relapse rates [34]. Similarly, another trial following the same busulfan administration schedule focused on patients aged 60 years or older or with comorbidities (AML, MDS, or myelofibrosis) reported an NRM of 9.3% and OS of 80% at 3 years [35].

#### 2.1.3. To Radiate or Not to Radiate

Three metanalyses have compared Bu/Cy and Cy/TBI. Gupta et al. [36] in 2011 and Hartman et al. [37] in 1998 did not find significant differences in disease-free survival or OS between these regimens. Hartman did describe a higher incidence of VOD with Bu/Cy (OR 2.5). Gupta reported lower transplant-related mortality with Cy/TBI than oral Bu/Cy (RR 0.53) [36,37]. The third meta-analysis compared several TBI and non-TBI-based regimens and found no difference in OS. However, cyclophosphamide given along with a higher dose of TBI was associated with increased NRM [16]. While no data are available comparing “low toxicity” radiation-based regimens, such as TMLI with alkylator-based conditioning in the setting of AML, TMLI-based conditioning has been shown to have favorable outcomes compared to non-TMLI regimens in acute lymphoblastic leukemia. A study comparing TMLI vs. non-TMLI conditioning in 70 adult patients with ALL reported an incidence of relapse of 25% vs. 46.5%, *p* = 0.018, and an overall survival of 73.1% vs. 52.6%, *p* = 0.033, favoring the TMLI group [38].

### 2.2. Reduced-Intensity Conditioning

Reduced-intensity conditioning was developed based on the premise that the graft-versus-leukemia effect can be enough to cure the malignancy. It is tailored towards effecting immunosuppression to allow engraftment while reducing toxicity. The two most common RIC regimens for AML are Fludarabine/Melphalan (Flu/Mel) and Fludarabine/Busulfan (Flu/Bu2, busulfan ≤ 6.4 mg/kg). Slavin et al. described using fludarabine, anti-T-lymphocyte globulin, and busulfan at 8 mg/kg given over 2 days in 1998 for HSCT in patients with diverse hematologic malignancies. The disease-free survival at 14 months was 77.5% [39]. Flu/Mel was developed by the MD Anderson Cancer Center transplant group for patients considered poor candidates for conventional myeloablative therapy. In a retrospective study by Oran et al., there was no difference in OS between patients receiving 140 mg/kg or 180 mg/kg in combination with fludarabine [40]. A CIMBTR registry study, including 622 patients with AML who underwent HSCT, reported that Flu/Mel results in improved leukemia-free survival (LFS), long-term OS, and lower relapse rate compared to Flu/Bu2. In that study, Flu/Mel was associated with an increased NRM in the first 3 months post-transplant compared to Flu/Bu2 (HR 3.85, *p* < 0.001) [41]. With increasing contemporary use of antibody-drug conjugates and patients with increasing comorbidities making it to HSCT, the use of melphalan may provide a safer alternative when considering the risk of complications, such as SOS/VOD, with Busulfan-based conditioning regimens.

RIC is preferred in older patients and those with comorbidities who are not eligible for MAC due to lower toxicity and NRM. A propensity score analysis-based study showed that RIC with Flu/Mel 100 resulted in improved PFS compared to Flu/Mel 140 and myeloablative regimens, particularly in patients older than 65 years old or with inadequate performance status (HR 0.57, *p* = 0.013) [42]. However, several studies have associated RIC with a higher risk of relapse. BMT CTN 0901, a phase III randomized trial, recruited patients who planned to undergo HSCT and assigned them to either RIC or MAC arms. At 4 years following transplant, the total relapse mortality (TRM) was 25.1% for the MAC group compared to 9.9% for the RIC group, and an HR for relapse of 4.06, favoring MAC (*p* < 0.001). OS was improved in the MAC cohort (HR of 1.54, *p* = 0.03), contrary to prior randomized trials [4,43,44,45]. An analysis of a subset of patients from BMT CTN 0901 with available next-generation sequencing-based MRD status data suggests that the heightened risk of relapse associated with RIC may primarily apply to those with positive MRD. The HR for relapse between RIC and MAC in patients with MRD positivity was 6.38 (*p* < 0.001), favoring those who received MAC. In contrast, for MRD-negative patients, the HR was 1.78 (*p* = 0.21) [5]. Despite the limitations of RIC, it has been shown to lead to better outcomes compared to standard chemotherapy for AML. Mohty et al. conducted a study involving 95 patients with AML who were considered potential candidates for HSCT with RIC. The patients were stratified based on the availability of a donor; 25 patients were able to proceed with HSCT. The LFS rate for those who underwent transplantation was 62%, compared to 21% for those who did not [46].

Treosulfan has been recently approved by the FDA based on a phase 3 trial that found it non-inferior to Flu/Bu2 for HSCT in patients 50 years or older and considered at increased risk for treatment with myeloablative regimens. Treosulfan is an alkylating agent that does not undergo hepatic metabolism and does not require TDM [47].

Another emerging modality is using a radioactive isotope conjugated to a monoclonal antibody targeting a protein preferentially expressed in hematopoietic stem cells. Recently, results of an open, phase 3 trial using a conditioning regimen of Flu-TBI and I-apamistimab (anti-CD45 antibody conjugated to iodine-131) versus conventional care in patients with active relapsed/refractory AML, 55 years or older, have been reported. The trial allowed for crossover for patients allocated in the standard of care group to the I-apamistimab arm if they achieved an inadequate response. In the intention-to-treat analysis, the median OS was similar between groups (HR 0.99, *p* = 0.96). However, patients who received conditioning with I-apamistimab did achieve a longer EFS (HR 0.23, *p*< 0.0001). The results of the trial should be interpreted in the context of a high cross-over rate (57% patients in the conventional treatment were ultimately treated with I-apamistimab) [48].

## 3. Conditioning for Patients with Active or Refractory Disease

Standard practice is to proceed to allogeneic stem cell transplantation after achieving complete remission (CR). As such, patients with refractory disease or those who develop complications from induction are not eligible for HSCT and have a poor prognosis. Efforts to develop conditioning regimens that allow patients with active disease to proceed with HSCT are ongoing. A phase II trial of 74 patients with acute leukemia (AML *n* = 56 and ALL *n* = 18, 97% with detectable marrow blasts and 69% with induction failure) who received conditioning of TMLI plus etoposide/cyclophosphamide reported a day +30 CR of 92%, 2-year PFS and 2-year OS of 31.1% and 45.9% with an NRM at 2 years of 12% [49]. A clinical trial based on this regimen is ongoing (NCT03467386). Additionally, The Alliance Leukemia and the German Cooperative Transplant Study Group conducted a phase III trial in which patients not in CR underwent HSCT conditioning with FLAMSA or received salvage chemotherapy with cytarabine plus mitoxantrone prior to transplant. Non-inferiority could not be established, but there was no difference in LFS or OS between treatment groups at 4 years [50].

## 4. Conditioning Regimens for Second Allogeneic Stem Cell Transplants

A second stem cell transplant may be required in case of graft failure or disease relapse. There are limited data to guide the choice of conditioning regimen in this case. A recent large registry-based study of 1540 patients by the European Society for Blood and Marrow Transplantation on second transplantation for AML reported that using a myeloablative regimen was associated with better outcomes. They reported a decreased incidence of relapse (HR 0.7, *p*  <  0.001), improved OS (HR 0.82, *p*  =  0.02), improved LFS HR 0.79, *p*  =  0.002), without an increase in NRM (HR 1.1, *p*  =  0.47) [51]. Nevertheless, patients who are being considered for a second transplant may not be eligible for MAC or RIC regimens based on their performance status. Our approach considers the time from the first to the second transplant, the prior regimen (radiation or non-radiation based), and the patient’s performance status. In patients with good performance status and with high-risk disease, TMLI is favored. In those who have previously received radiation as a conditioning regimen, we use Flu/Mel as our preferred conditioning modality. Lastly, in those not eligible for MAC or Flu/Mel-based regimens, we prefer non-myeloablative regimens such as, Flu/Cy/TBI 2 Gy.

## 5. Conclusions

**Figure 2:**
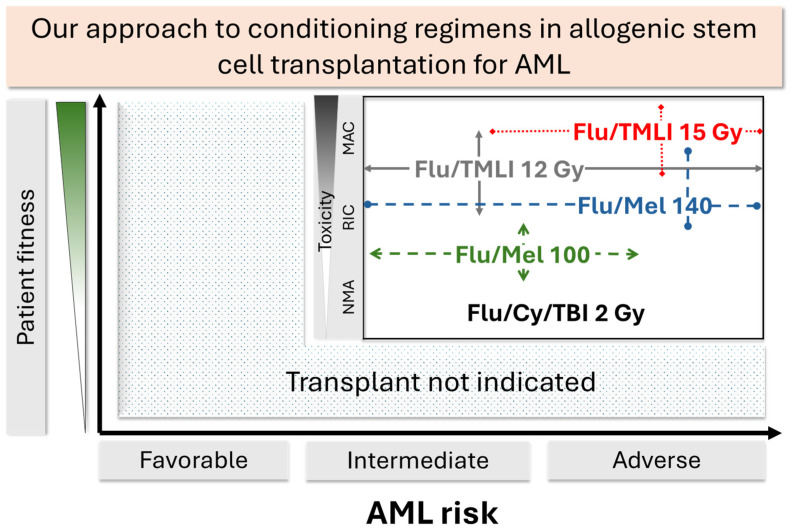
Our approach to allogeneic stem cell transplantation for acute myeloid leukemia. The choice of conditioning is based on AML risk, the patient’s fitness, and the expected toxicity of the regimen. This figure intuitively outlines the most common conditioning regimens used at our institution. The horizontal arrows represent the applicability of each conditioning regimen based on ELN 2022 risk, and the vertical arrows correlate with the expected regimen toxicity.

The optimal conditioning regimen for treating a patient with AML depends on several factors, including the risk level of the disease, the patient’s condition at the time of transplantation, their age, overall health, MRD, and local clinical experience. Current data do not indicate that chemotherapy is superior to radiation-based treatments; instead, the choice of conditioning often relies on institutional expertise (Figure 2). New treatment methods have made it possible to escalate doses for patients who may not have qualified for myeloablative conditioning, often leading to better disease control. Furthermore, advancements in these regimens are allowing a broader range of patient populations to benefit from hematopoietic stem cell transplantation due to reduced toxicity. Innovations that allow for the reduction of toxicity while maintaining the anti-leukemic effects of conditioning are expected to continue improving outcomes.

## Figures and Tables

**Table 1:** Average Organ Dose (Gy) as a percentage of the prescribed target dose for one of our patients.

Organs	Percent of the Prescribed Target Dose
Bowel	70.91%
Brain	71.00%
Esophagus	48.24%
Eyes	56.78%
Lens	56.27%
Heart	53.06%
Kidneys	62.33%
Larynx	56.85%
Liver	60.00%
Lungs	58.83%
Oral Cavity	29.25%
Parotids	71.37%
Rectum	87.64%
Thyroid	89.65%

## Abbreviations

The following abbreviations are used in this manuscript:

AML	Acute myeloid leukemia
Bu/Cy	Busulfan, cyclophosphamide
Bu/Flu	Busulfan, fludarabine
CIBMTR	Center for International Blood and Marrow Transplant Research
CR	Complete remission
Cy/TBI	Cytarabine, total body irradiation
FLAMSA	Fludarabine, cytarabine, amsacrine
Flu/Mel	Fludarabine, melphalan
HR	Hazard ratio
HSCT	Hematopoietic stem cell transplantation
LFS	Leukemia-free survival
MAC	Myeloablative conditioning
MDS	Myelodysplastic syndrome
NMA	Nonmyeloablative
NRM	Non-relapse mortality
OS	Overall survival
RFS	Relapse-free survival
RIC	Reduced intensity conditioning
TBI	Total body irradiation
TDM	Therapeutic drug monitoring
TMI	Total marrow irradiation
TMLI	Total marrow and lymphoid irradiation
TRM	Transplant-related mortality
VOD	Veno-occlusive disease
